# Sex-Associated Expression of Co-Stimulatory Molecules CD80, CD86, and Accessory Molecules, PDL-1, PDL-2 and MHC-II, in F480+ Macrophages during Murine Cysticercosis

**DOI:** 10.1155/2013/570158

**Published:** 2013-01-20

**Authors:** Cristián Togno-Peirce, Karen Nava-Castro, Luis Ignacio Terrazas, Jorge Morales-Montor

**Affiliations:** ^1^Departamento de Inmunología, Instituto de Investigaciones Biomédicas, Universidad Nacional Autónoma de México, AP 70228, 04510 México, DF, Mexico; ^2^Departamento de Biología, Facultad de Química, Universidad Nacional Autónoma de México, 04510 México, DF, Mexico; ^3^Unidad de Biomedicina, Facultad de Estudios Superiores-Iztacala, Universidad Nacional Autónoma de México, Avenida de Los Barrios No. 1 Los Reyes Iztacala, 54090 Tlalnepantla de Baz, MEX, Mexico

## Abstract

Macrophages are critically involved in the interaction between *T. crassiceps* and the murine host immune system. Also, a strong gender-associated susceptibility to murine cysticercosis has been reported. Here, we examined the sex-associated expression of molecules MHC-II, CD80, CD86, PD-L1, and PD-L2 on peritoneal F4/80^hi^ macrophages of BALB/c mice infected with *Taenia crassiceps*. Peritoneal macrophages from both sexes of mice were exposed to *T. crassiceps* total extract (TcEx). BALB/c Females mice recruit higher number of macrophages to the peritoneum. Macrophages from infected animals show increased expression of PDL2 and CD80 that was dependent from the sex of the host. These findings suggest that macrophage recruitment at early time points during *T. crassiceps* infection is a possible mechanism that underlies the differential sex-associated susceptibility displayed by the mouse gender.

## 1. Introduction

Gender of the host influences the outcome of many parasitic diseases. For example, in *Leishmania major* infection, female mice mount a strong Th1 response and resolve the infection. In contrast, male mice mount a Th2-dominant response and develop chronic lesions [1]. In other protozoan infections, such as toxoplasmosis, an opposite finding was observed: female mice succumb to *Toxoplasma gondii* infection despite a Th1 response, whereas male mice display resistance and survive for a longer period of time to similar challenges [2].

In helminth infections, the gender of the host also plays an important role in the outcome of the infection by inducing different responses depending on the sex [3, 4]. In contrast to the well-described adaptive immunity against these helminthic infections, the role of macrophages (M*ϕ*s) is still unclear. There have been only limited studies on the macrophage response to helminth-derived antigens and the impact of these responses on the outcome of the infection is not known. Much lesser information exists in relation to the role of sex on the macrophage response to helminth-derived antigens.

A sexual dimorphism exists in the acquired immune response against different pathologies and in many autoimmune diseases, which suggests a linkage between the immune and reproductive endocrine system [5]. Moreover, reciprocal endocrine interactions between host and parasite are a strong factor that has an influence in parasite success [6, 7]. 

Experimental murine cysticercosis caused by *Taenia crassiceps* [8, 9] is well known as a manageable experimental system which explores the role of biological factors involved in host susceptibility [10]. Interestingly, in *T. crassiceps* cysticercosis, females of all strains of mice studied sustain larger intensities of infection than males [11]. At the same time, the cellular immune response (Th1) is markedly diminished in both sexes, and the humoral response is enhanced (Th2) [12]. Estradiol is involved in the immunoendocrine regulation of murine *T. crassiceps* cysticercosis as a major protagonist in promoting cysticercus growth by interfering with the thymus dependent cellular immune mechanisms that obstruct parasite growth [13]. Gonadectomy alters this resistance pattern and makes intensities equal in both sexes by increasing that of male mice and diminishing it in female mice [14]. In addition, the hormonal substitution of gonadectomized males and reconstitution of female mice with 17*β*-estradiol increased parasite loads [13]. Also, specific splenocyte cell proliferation, IL-2, and IFN-*γ* production were depressed in gonadectomized-parasitized mice of both genders, and after the reconstitution with testosterone or dihydrotestosterone, there was a significant recovery of the splenocyte proliferation and Th1 cytokine production on these animals. On the other hand, mice treated with estradiol were not able to induce these cellular responses [15].

Macrophages are phagocytic cells that are widely distributed on the organism and have an important role in the maintenance of the homeostasis [16]. These cells are involved in T cell activation through antigen presentation by the expression of MHC molecules and costimulatory/inhibitory molecules. It has been demonstrated that the expression of MHC molecules and the expression of costimulatory molecules such as B7-1 (CD80) and B7-2 (CD86) could modulate T cell activation and Th1/Th2 polarization during infection and autoimmunity [17, 18]. Programmed death ligand 1 (PD-L1) and programmed death ligand 2 (PD-L2) have been related to alternate activated phenotype in macrophages induced during *Taenia crassiceps* infection [19]. Macrophages also have a broad participation in the development of the immune response to many pathogens, particularly to helminthes [20] by polarizing T helper (Th) cells activation in Th1 or Th2, and also have a role in tissue remodeling and wound repair [21]. In the context of immunoendocrine communication, it has been shown that sex steroids are able to modulate survival of human macrophages cell lines [22], the recruitment of macrophages to the site of inflammation, and their effector functions [23]. As occurred with other immune cells, the effect of sex steroids on macrophages depends on the concentration, type, and the context in which macrophages are studied [24]. Furthermore, it has been previously established that sex steroid effects on macrophages depend on the expression of the androgen receptor (AR) [25, 26], progesterone receptor (PR) [27], and both types of estrogen receptor (ER*α* y ER*β*) [28]. 

Since macrophages have been importantly involved in susceptibility/resistance in murine cysticercosis and also can be modulated by sex steroids, we evaluated and compared the response of molecules of early activation of recruited F4/80^hi^ macrophages, such as MHC-II, CD80, CD86, PD-L1, and PD-L2 in both gender infected mice. Our results showed that indeed there is a differential expression of these molecules in female and male mice and that this could partially impact the different sex-associated susceptibility to cysticercosis in mice.

## 2. Materials and Methods

### 2.1. Ethics Statement

The Animal Care and Use Committee at the Instituto de Investigaciones Biomédicas evaluated animal care and experimentation practices according to the official Mexican regulations (NOM-062-ZOO-1999) in strict accordance with the recommendations in the Guide for the Care and Use of Laboratory Animals of the National Institutes of Health (NIH and the Weatherall Report) of the USA. The Ethics Committee of the Instituto de Investigaciones Biomédicas approved this protocol (Permission Number 2009-13). 

### 2.2. Animals and Experimental Infections

Male and female BALB/cAnN (H2-d) inbred mice obtained from Harlan (Mexico City) were used in all experiments. Animals were housed in the animal care facilities at Instituto de Investigaciones Biomédicas, (UNAM), under controlled conditions of temperature and 12 h dark-light cycles with lights on between 0700 and 1900. They were fed Purina Diet 5015 (Purina, St. Louis, MO) and given tap water ad libitum. 

### 2.3. Aantigen Extraction and Infection

Metacestodes of *Taenia crassiceps *of the ORF strain were harvested in aseptic conditions from the peritoneal cavity of female BALB/cAnN mice after 4 months of infection. Metacestodes were washed with cold sterile saline (Solución CS, Laboratorios PISA. S.A. de C.V. [NaCl 0.9%]). *T. crassiceps *soluble extract (TcEx) was prepared in cold aseptic conditions, homogenizing whole metacestodes (30 mL volume) with tree pulses of 60 Hz with a duration of 1 s, by using an ultrasonic homogenizer (Vibracell, SONICS & MATERIALS, Newtown, USA). The homogenates were centrifuged at 20,000 g for 30 min at 4°C, and the supernatants containing saline-soluble antigens were collected and frozen at −20°C until further use. Protein concentration was estimated by Bradford protein kit assay (BioRad). Sex-and age-matched mice were infected by intraperitoneal (ip) injection with 20 small (approximately 2 mm) nonbudding cysticerci/300 *μ*L saline, with 400 *μ*g TcEx in 300 *μ*L saline or 300 *μ*L saline as control. Six days after-injection, animals were sacrificed by inhalation of an overdose of sevoflurane (Sevorane; Abbott) and peritoneal cells were collected for analysis.

### 2.4. Isolation of Peritoneal Macrophages

Peritoneal exudate cells (PECs) were obtained from saline, TcEx-treated, or 6-day-*T. crassiceps* infected mice (BALB/c male or female) by peritoneal lavage with 7 mL of sterile ice-cold saline (Laboratorios PISA. S.A. de C.V. [NaCl 0.9%]). The cells were washed twice with cold PBS. After two washes, the viable cells were counted by trypan blue exclusion with a Neubauer hemocytometer. Viable cells were counted and adjusted to 1 × 10^6^ cells/mL. Viability was measured by trypan blue exclusion. Routinely viability was around over 95%. 

### 2.5. Analysis of Cell Surface Markers in Macrophages

The surface expression of macrophage markers was analyzed using multicolor flow cytometry. M*ϕ*s were suspended in cold PBS containing 2% FCS and 0.02% NaN_3_. The Fc receptors were blocked with anti-mouse CD16/CD32 for 20 min at 4°C. The cells were washed and triple stained with an APC-conjugated mAbs against F4/80, PE-conjugated mAbs against CD86 or PD-L2, PerCP-conjugated IA/IE (MHC-II), PE-Cy5-conjugated CD80 or Biotin-conjugated PD-L1, and PE-Cy5-conjugated Streptavidin. All Abs were purchased from BioLegend (BioLegend, San Diego, CA, USA). A gate including high forward light scatter (FSC)/high side light scatter (SSC) cells was generated and in that gate the different markers were analyzed. The stained cells were captured using a FACsCalibur flow cytometer (Becton Dickinson) and data analyzed with the FlowJo (Tree Star) software. Absolute numbers in all assays were calculated according to the percentage of positive macrophages and the total numbers of PECs.

### 2.6. Statistical Analysis

The data of the three replications of each experiment were pooled and expressed as their average. The data were analyzed using analysis of variance (ANOVA) with sex (2 levels) and number of experiment (3 levels) as independent variables and as dependent variables: the total number of developed cysticerci and the expression of each molecule. If significant differences between treatments were found by ANOVA, differences between the group means were assessed within each experiment by means of Tukey test using the residual variance estimated by ANOVA to test for significance. Differences were considered significant when *P* < 0.05.

## 3. Results

**Figure 1:**
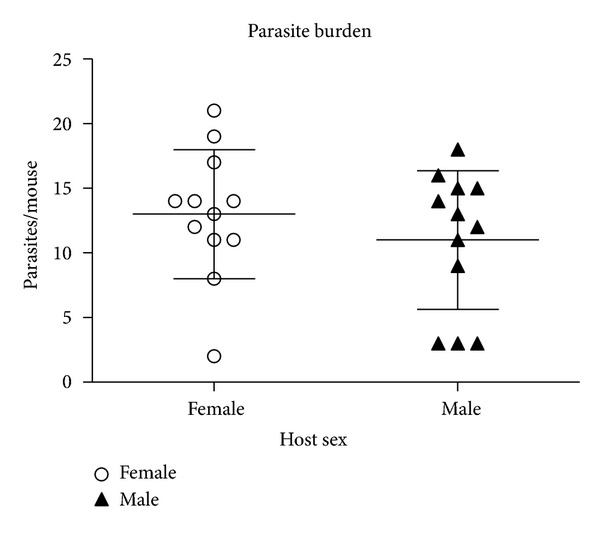
Parasite load obtained of Female (F) and male (M) mice. Data show the number of parasites recovered from the peritoneal cavity at 6 days post-infection. At this time of infection, animals did not show the typical sexual dimorphism of this infection observed in longer infection times. Each point represents individual parasite loads.

In order to determine the role of sex during early infection, mice of both sexes were infected and sacrificed 6 days after-infection. As previously reported [29], at this time point of infection, there is no statistical difference in parasite loads between males and females, though there is a slight trend in males to have less parasites than females (Figure 1). This result is also consistent with the observation that sexual dimorfism begins after the first week of infection in BALB/c mice [30].

**Figure 2:**
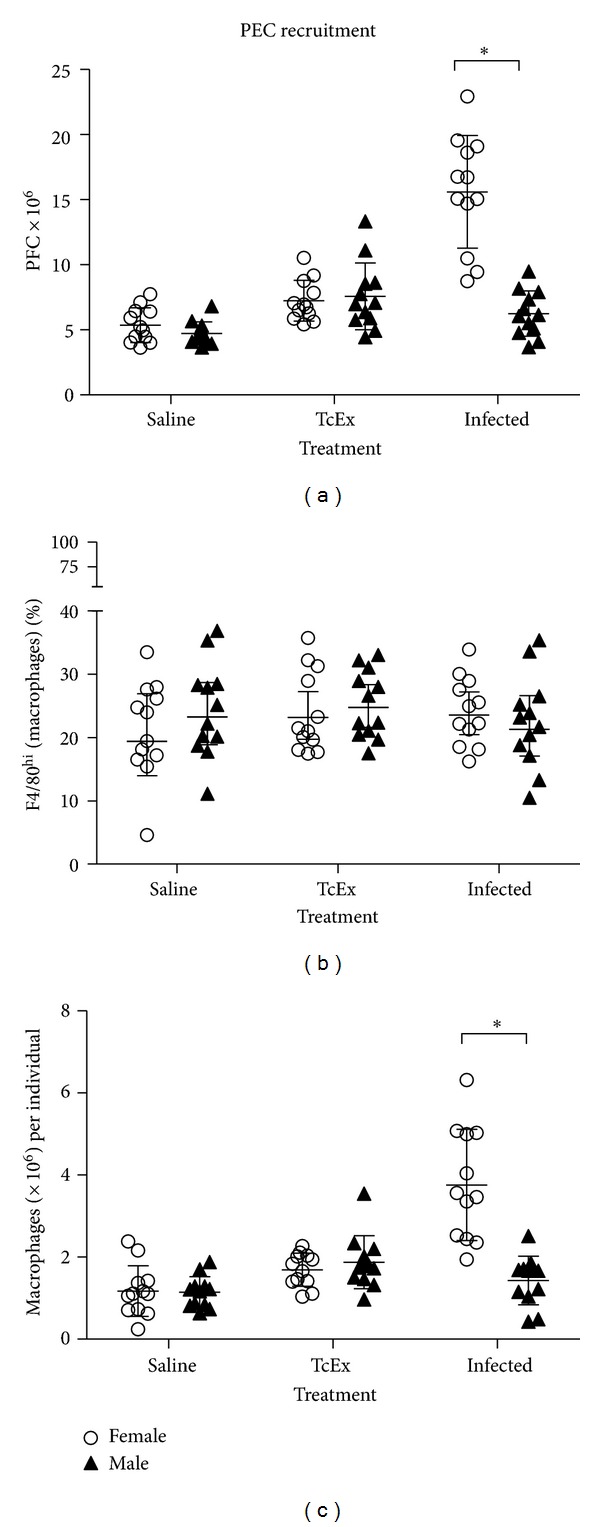
Kinetics of total peritoneal exudate cells (PECs) recovered from the peritoneum after *T. crassiceps* infection. (a) Peritoneal cells were isolated from male and female BALB/c at 6 days after infection with twenty cysticerci. Without any additional stimulation, the cells were processed for flow cytometry and analyzed. (b) Flow cytometry analysis shows that macrophages (F4/80^hi^) are recruited within 6 days p.i. (d.p.i.). (c) Increased numbers are detected per individual associated to sex as infection progresses peritoneal exudate cells.

To detect the presence of M*ϕ*s and to look for a difference in the number of total M*ϕ*s during early infection, we analyzed the population of PECs recruited to the peritoneal cavity (site of infection) of saline-treated, TcEx, and infected mice of both sexes. Total PECs recruitment in infected male mice is decreased (*P* < 0.05) compared to infected females, while treatment with saline solution or TcEx did not affect the total number of PECs recruited (Figure 2(a)). Since M*ϕ*s have been previously involved in the susceptibility/resistance to murine cysticercosis, we decided to analyze their percentage (Figure 2(b)) and their total number (Figure 2(c)), defined by their high expression of F4/80 (F4/80^hi^). We found no differences in the percentage of F4/80^hi^ cells between sexes (Figure 2(b)), but there was a marked increase in the total number of M*ϕ*s detected in infected females with respect to infected males. This difference was not observed in the other treatments (Figure 2(c)). 

**Figure 3:**
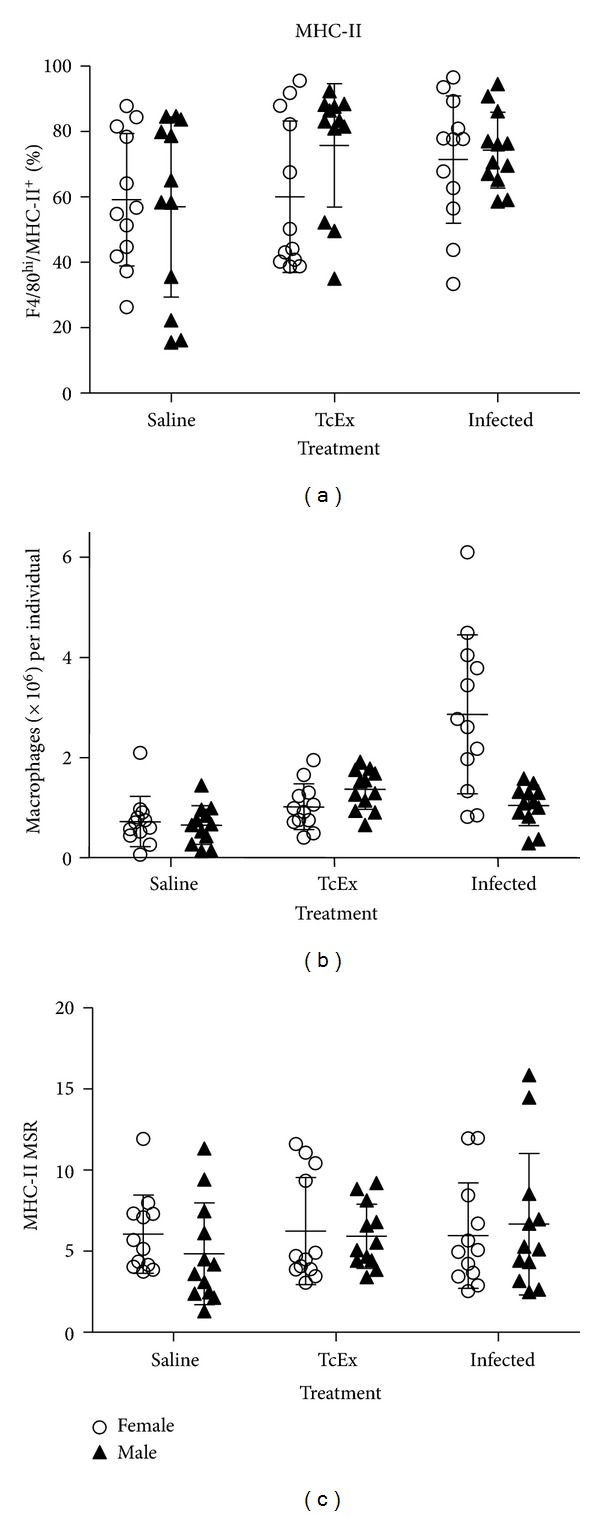
MHC-II characterization in M*ϕ*s recovered from the peritoneum after *T. crassiceps* infection. MHC-II expression was determined on M*ϕ*s (F4/80^hi^) recruited after 6 days of infection with 20 cysts of *T. crassiceps*. The percentage (a), total numbers (b), and the expression of this molecule (relative mean intensity, MSR) (c) are shown.

**Figure 4:**
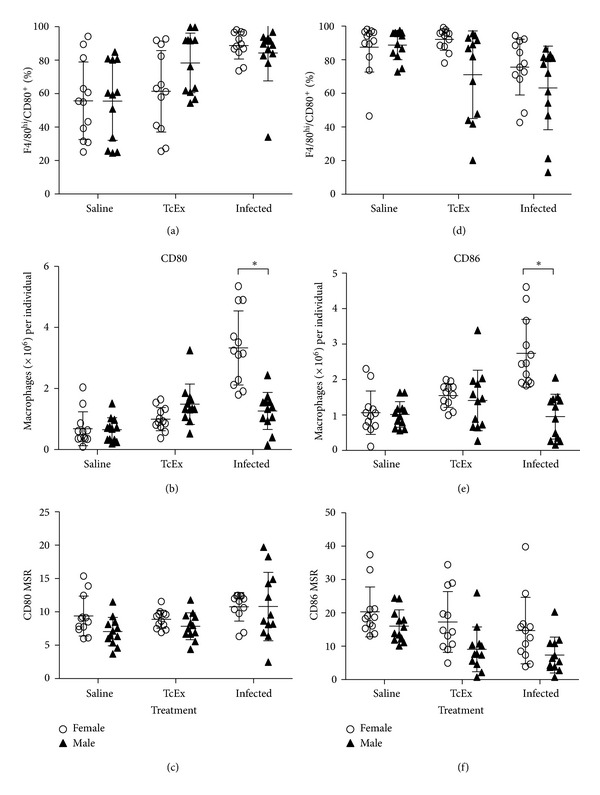
CD80/CD86 characterization in M*ϕ*s recovered from the peritoneum after *T. crassiceps* infection. Costimulatory molecules CD80 and CD86 expression was determined on M*ϕ*s (F4/80^hi^) recruited after 6 days of infection with 20 cysts of *T. crassiceps*. The percentages (a) and(d), Total numbers (b) *and *(e), and the expression of these molecule (relative mean intensity, MSR) (c) and (f) are shown.

**Figure 5:**
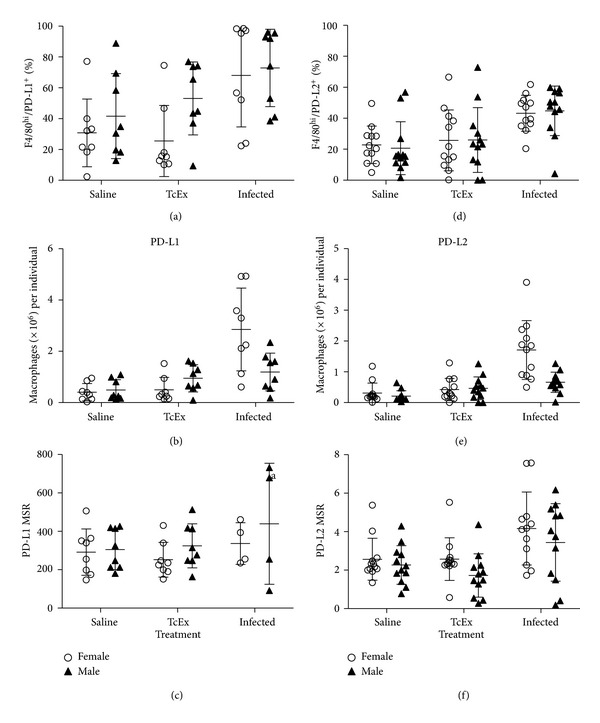
PD-L1/PD-L2 characterization in M*ϕ*s recovered of the peritoneum after *T. crassiceps *infection. Inhibitory molecule, PD-L1, and PD-L2 expression was determined on M*ϕ*s (F4/80^hi^) recruited after 6 days of infection with 20 cysts of *T. crassiceps*. The percentages (a) and (d), total numbers (b ande), and the expression of these molecules (relative mean intensity, MSR) (c) and (f) are shown.

To characterize the phenotype of M*ϕ*s recruited of the peritoneal cavity of infected mice of both sexes, we look for the expression of MHC-II (Figure 3), CD80/CD86 (Figure 4), and PD-L1/PD-L2 (Figure 5) by flow cytometry. In Figure 3(a), the percentage of MHC-II+ cells found is depicted. There is no difference associated to treatment or sex, in the percentage of M*ϕ*s expressing these molecule. However, as seen in the total number of PECs of infected female mice, the total number of M*ϕ*s MHC-II+ is also increased (Figure 3(b)). As for the relative mean intensity of the expression of MHC-II (a measure of the amount of the total MHC-II per cell), there is no difference between animals, either by treatment or sex (Figure 3(c)).

In Figure 4, the analysis of the expression of CD80 and CD86 is plotted. There were no differences associated to sex in the percentage of M*ϕ*s expressing CD80 or CD86 (Figures 4(a) and 4(d)). However, there is a marked difference in the total number of CD80+ or CD86+ M*ϕ*s that is observed in infected mice; female mice show an increased number of this population when compared to male mice (Figures 4(b) and 4(e)). We also compared the relative mean intensity (MSR) of the expression of these molecules, in terms to define differences in the coestimulatory ability of these cells. We found no differences between male and female mice in terms of CD80 expression either by treatment or sex (Figure 4(c)). However, CD86 appeared less expressed in male mice than in female mice, even when these data did not show significance (Figure 4(f)).

Finally we look for differences in the expression of PD-L1 and PD-L2 inhibitory molecules. As shown in Figures 5(a) and 5(d), there were no differences in the percentage of PD-L1 or PD-L2 expressing M*ϕ*s among males and females, but there were differences among treatment: infection induced a higher expression of PD-L1/2 than TcEx. As observed for CD80/86, there were also higher numbers of PD-L1- or PD-L2-positive M*ϕ*s in infected female mice than in infected male mice (Figures 5(b) and 5(e)); but there were no changes in the expression of these molecules on M*ϕ*s (Figures 5(c) and 5(f)). 

## 4. Discussion

Given the reported importance of sex- and pregnancy-associated hormones in the establishment and outcome of parasitic diseases, this is an area of research that is likely to grow. The important role that sex steroids plays during murine cysticercosis has been previously demonstrated in experiments in which gonadectomy, thymectomy, and whole body irradiation showed that both the endocrine and immune systems of the mice were involved in the parasite load differences between the host sexes. Interestingly orchidectomy in male mice raises parasite loads while ovariectomy has the opposite effect; it increased them 3-fold [14]. Thymus hindered parasite reproduction in both sexes but more so in males than in females, thus tending to equalize the number of parasites in thymectomized mice of both sexes [31]. 

Macrophages play a key role in directing the host immune response to parasites and they can also function as effector cells. The recruitment and activation of macrophages by helminth-derived molecules initiate with the expression of accessory molecules. These immune mediators play crucial roles in the development of immunity against a variety of pathogens, but their role in helminthic infections is less well understood [32, 33]. In this study, we found an increased number of recruited macrophages from *T. crassiceps*-infected female BALB/c mice in comparison with male mice and expressed MHC-II, the coestimulatory molecules CD80, CD86, and the accessory molecules PD-L1 and PD-L2. However, the major difference that we found was associated to infection, though a clear difference in the number of parasites did not exist. There were more M*ϕ*s in infected females compared to those observed in infected males after similar stimulation. These data are consistent with the susceptible phenotype observed in IL-12 KO mice [34] and suggest a major role for macrophages in cysticercosis. The mechanism underlying the differential expression of MHC-II, CD80, CD86, PD-L1, and PD-L2 in our system remains to be elucidated; however, it may be associated with an impaired intracellular signaling in BALB/c male mice but not in female mice.

The relevance of these observations is highlighted by the finding that macrophages from BALB/c female mice became more rapidly alternatively activated in *T. crassiceps* chronic infection, whereas macrophages from male mice presented a transient and incomplete alternate activation during early infection [35]. Thus, the presence and the persistence of AAM*ϕ* are another striking difference between the susceptible and resistant sex of mice to *T. crassiceps* infection. 

In the context of immunoendocrine communication, it has been previously established that macrophages express the androgen receptor (AR) [25, 26], progesterone receptor (PR) [27], and both types of estrogen receptor (ER*α* y ER*β*) [28]. It has been shown that sex steroids are able to modulate survival of human macrophages cell lines [22], the recruitment of macrophages to the site of inflammation [23], and their effector functions. As occurred with other immune cells, the effect of sex steroids on macrophages depends on the concentration, type, and the context in which macrophages are studied [24].

For instance, in the murine model of incisional wound, gonadectomy of females is associated to an increased inflammation and delay in wound healing. This effect is due to the fact that ovariectomy induces an increase in the secretion of TNF-*α* and MIF, as well as in the number of infiltrated macrophages at the site of the lesion. Also, the percentage of alternatively activated macrophages is decreased [23, 36]. If castrated females are reconstituted with E2 concentrations observed during estrous, then the production of TNF-*α*, MIF, and the total number of infiltrated macrophages in the wounds are decreased. However, treatment with physiological levels of progesterone has a modest effect, in comparison to the effect induced by estradiol, on the same parameters studied [23]. Moreover, sex steroides regulate the production of nitric oxide (NO) by macrophages, in a dichotomic way. At low concentrations, E2 stimulates the secretion of NO by LPS-activated macrophages in vitro; however, at high concentrations of E2, there is a decrease of NO [37, 38]. Furthermore, estradiol and to a lesser extent progesterone decrease the activity of the enzyme catalase, a very important modulator of the NO synthesis [39]. As such, these data may represent an important mechanism underlying the immunomodulating effects of sex steroids. 

Previously, we showed that during murine cysticercosis, an impressive feminization process is produced in the male host, characterized by an increase in serum estradiol level of 200 times above their normal value, roughly similar to those of normal females, while those of testosterone decreased by 90% relative to controls [29]. These changes in the hormonal milieu of the host equalize the parasite loads between genders. In the same way, progesterone treatment tends to equalize parasite loads in females and males, which suggests that other gonad-associated factors are involved in the control of parasite growth. Therefore, a more intricate strategy of parasite activity has to be considered. Perhaps, high estrogen levels are the main feature of this intriguing puzzle, since, in males, the parasite loads increased more markedly than in females. We suppose that expression of costimulatory molecules early during infection could be differential, and this fact impacts the parasite loads that are different among males and females, late during infection. This hypothesis was tested in this study and found that always females have higher expression of MHC-II, CD80, CD86, PD-L1, and PD-L2 during infection, but not in response to saline or TcEx. Interestingly, estradiol concentrations are higher in infected females early in infection [29]. 

## 5. Conclusion

In summary, the results presented here demonstrate that recruitment and expression of MHC-II, CD80, CD86, PD-L1, and PD-L2 in M*ϕ* of peritoneal cavity in *T. crassiceps* early at infection is associated to the sex of the host, although at the time of infection the number of parasites does not differ between both sexes. Whatever the cysticercosis-relevant “sex steroid target” may prove to be, the fact steroids positively may interfere with the development of protective immune mechanisms against* Taenia crassiceps* cysticerci has an important implication for future vaccine and vaccination trials, among others projections.
